# Manipulative Ability

**Published:** 1894-05

**Authors:** 


ARTICLE III.

### MANIPULATIVE ABILITY.

Some dentists never become good gold fillers. They try after a fashion, but "it will not stick." They observe how easily an expert does it, but they can not imitate him. Their fillings will slip out, their best work will not last. It is bound to develop some defect somewhere.

Some time since we were in the office of a celebrated plastic filler when he advocated cement for the front teeth.

"But why not fill them with gold?" inquired the lady.

"The teeth are too frail," was the reply.

"Are they worse than most decayed teeth?"

"No; but in most decayed teeth cement is better, and looks better."

"Will it last as well?"

"Neither is permanent; all fillings need renewing at stated times; and though cement may need renewing oftener, it is a preserver of the teeth where gold is not."

We were astonished. But when, in after years, this dentist's poor gold fillings came under our inspection, we found he was a poor manipulator of gold.

We were in the office of a popular dentist some time since, when the operator said to another dentist:

"Doc, I have filled this small cavity with gold three times within the last three hours, and the filling will not stick. Let me see how you would operate."

In fifteen minutes it was filled; when the first said:

"Now, will you be offended if I just pick it out to test its hold?"

"No; but you can not pick it out without cutting it out bit by bit."

True enough; repeated trials showed that no ordinary force would remove it.

The difference cannot be easily explained. It was a something in the finger tips that did it. The gold was the same, and the cavity was unchanged. It was wholly in the manipulation.

But though the majority of dentists are poor gold fillers, most may attain skill by *doing their best every time*. The motto with us all should be, *Quality first, then speed*, but quality at all expense of speed or any other consideration. Better have the reputation of being a slow but sure workman than a poor one.

We had a student once who became a good gold filler, but could never hurry at this work, or any other. It was sure to spoil anything he did. As he was forty-five years of age, we despaired of his ever becoming alert and expert enough to attain distinction. But after a few years in his own office he became quite popular, for he gained the reputation of being an excellent dentist—though slow.
—EDITORIAL in *Items of Interest*.

